# Supplementary material for: Whole body PD-1 and PD-L1 positron emission tomography in patients with non-small-cell lung cancer
Source: Nat Commun. 2018 Nov 7;9:4664. doi: 10.1038/s41467-018-07131-y (PMC6220188; doi:10.1038/s41467-018-07131-y)
Supplement: Supplementary file 1 — Supplementary Information [file 41467_2018_7131_MOESM1_ESM.pdf]

Supplementary material

Niemeijer et al.

Supplementary Figure 1.

a

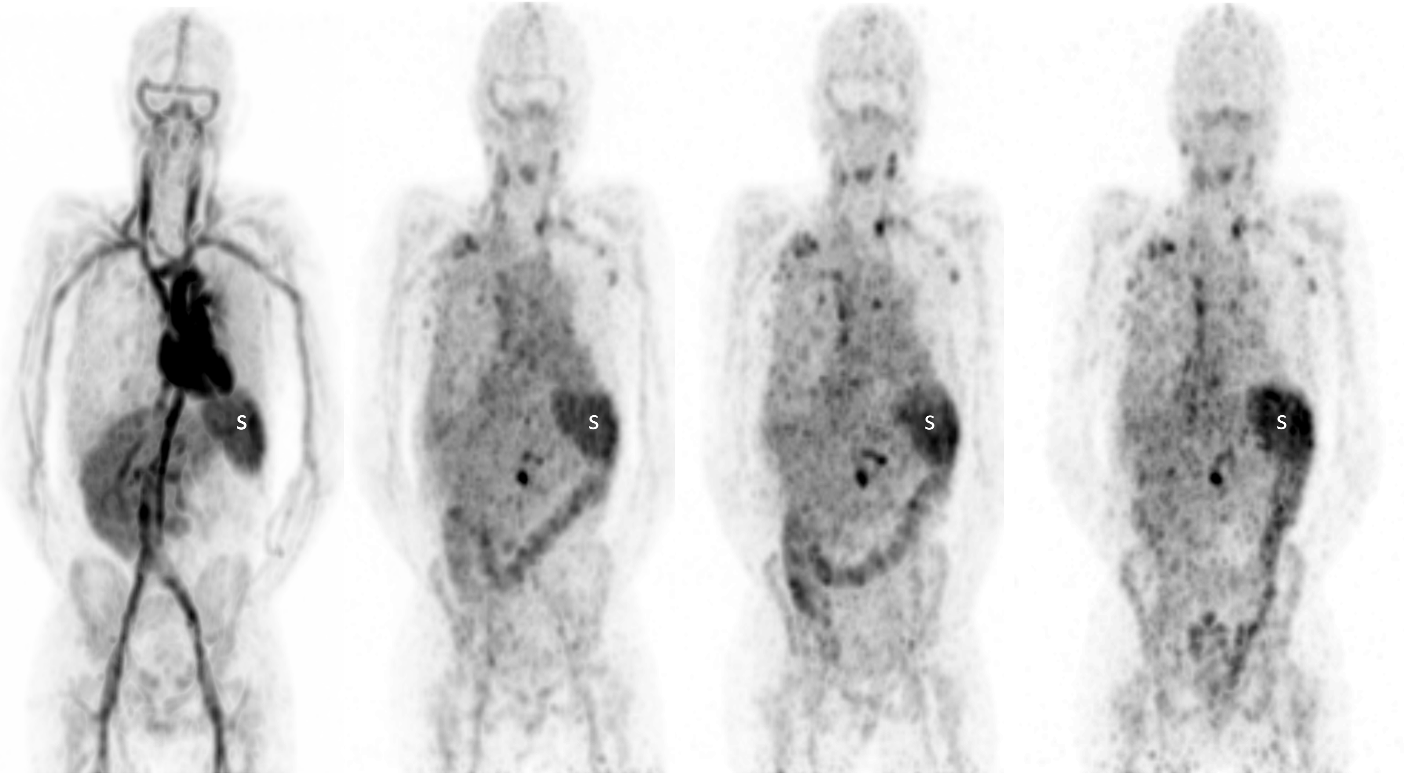

b

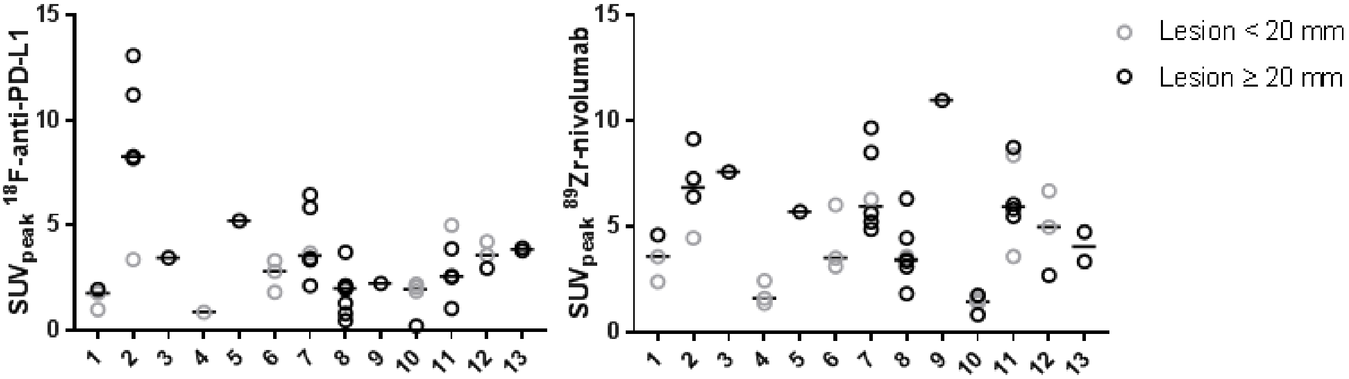

Supplementary Figure 1. Biodistribution and tumor uptake per lesion per patient. *a* ^{89}Zr-Nivolumab distribution on day 0, 3, 5 and 7 for patient 2. S = spleen. *b* SUV_{peak} of all lesions detected for the ^{18}F-BMS-986192 and ^{89}Zr-nivolumab tracer. In *b*, the bars represent the median.

Supplementary Figure 2.

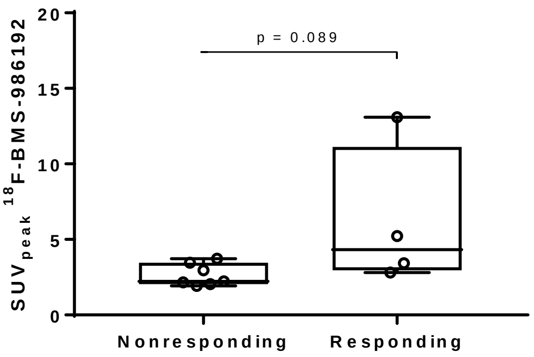

Supplementary Figure 2. Tumor uptake and response. SUV_{peak} of the ^{18}F-BMS-986192 tracer is higher in biopsied responding lesions compared to biopsied not biopsied lesions. P-value is 0.089 as determined by the Mann-Whitney-Wilcoxon rank sum test. In the boxplot, the lower edge of the box represents the first quartile and the upper edge represents the third quartile. The horizontal line inside the box indicates the median. Whiskers identify the minimum and the maximum value.

Supplementary Table 1

**Baseline characteristics**	
Median age, years	63.0 (58.0-69.0)
**Sex**	
Male	7 (53.8)
Female	6 (46.2)
**Histology**	
Adenocarcinoma	9 (69.2)
Squamous cell carcinoma	3 (23.1)
NOS	1 (7.7)
**WHO performance status**	
0	5 (38.5)
1	8 (61.5)
**Tumor PD-L1 expression**	
< 1 %	6
≥ 1 %	7
≥ 50%	2
**PD-1 expression in aggregates**	
IC0	6 (46.2)
IC1/IC2	7 (53.8)
**Number of previous anticancer regimens**	
0	1 (7.7)
1	7 (53.8)
2	4 (30.8)
> 2	1 (7.7)
Untreated CNS metastases	2 (15.4)

Supplementary Table 1. Baseline characteristics.

Supplementary Table 2

Adverse events	Grade I/II	Grade III/IV
**Gastrointestinal**		
Nausea	1 (7.7)	
**General**		
Anorexia	3 (23.1)	
Fatigue	4 (30.8)	
Headache	1 (7.7)	
Obstipation	2 (15.4)	
Pain	1 (7.7)	
**Injury**		
Hip fracture		1 (7.7)
**Laboratory abnormalities**		
Anemia	7 (53.8)	
Amylase increased	1 (7.7)	
ALP increased	1 (7.7)	
ALT increased	1 (7.7)	
AST increased	1 (7.7)	
Hyperkalemia	1 (7.7)	
Hypoalbuminemia	1 (7.7)	
Hyponatremia	1 (7.7)	
GGT increased	1 (7.7)	
Lipase increased		1 (7.7)
**Respiratory**		
Cough	1 (7.7)	
Dyspnea	2 (15.4)	

Supplementary Table 2. Adverse events upon injection tracers. ALP: Alkaline phosphatase. ALT: Alanine transaminase. AST: aspartate transaminase. GGT: gamma-glutamyltransferase

Supplementary Table 3

**Organs**	**SUV$_{mean}$** ^{89}**Zr-nivolumab** without predose	**SUV$_{mean}$** ^{89}**Zr-nivolumab** with predose	**SUV$_{mean}$** ^{18}**F-BMS-986192**
Spleen	5.8 ± 0.7	3.5 ± 0.9	15.7 ± 4.0
Liver	4.8 ± 2.2	4.9 ± 1.6	6.0 ± 1.8
Lung	1.3 ± 0.4	1.3 ± 0.2	1.5 ± 0.4
Kidney	2.8 ± 0.7	2.9 ± 0.5	20.5 ± 8.2
Bone marrow	2.5 ± 0.7	2.1 ± 0.6	3.2 ± 1.0
Brain	0.5 ± 0.1	0.6 ± 0.1	0.2 ± 0.04
Gall bladder	-	-	25.1 ± 11.0
Bladder	-	-	27.9 ± 16.9

Supplementary Table 3. **Biodistribuition in SUV$_{mean}$ per organ.**

Supplementary Methods

Synthesis of $[^{18}F]$BMS-986192

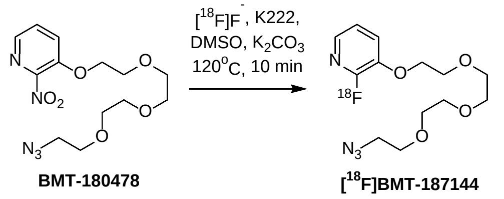

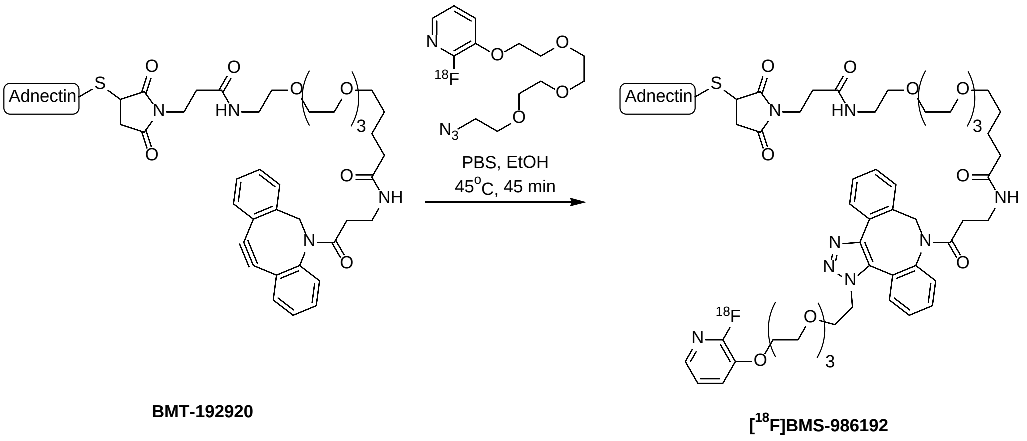

Production of [18F]BMS-986192: $[^{18}F]F^-$ was produced by the $^{18}O(p,n)^{18}F$ nuclear reaction using an IBA (Louvain-la-Neuve, Belgium) Cyclone 18/9 cyclotron. Radioactivity levels were measured using a Veenstra (Joure, The Netherlands) VDC-405 dose calibrator. Radiochemistry was carried out in home-made, remotely controlled synthesis units. After irradiation, $[^{18}F]F^-$ was trapped on a PS-HCO_3^- column (ABX, Radenberg, Germany). It was eluted from the anion exchange column into a screw cap reaction vessel with 1 mL of acetonitrile/water (9/1, v/v) containing Kryptofix 2.2.2 (13.0 mg, 34.6 µmol, 4,7,13,16,21,24-hexaoxa-1,10-diazabicyclo[8.8.8]hexacosane) and potassium carbonate (2.0 mg, 15 µmol). The solution was evaporated to dryness under a helium flow (50 mL/min−1) and reduced pressure at 90 ºC. Acetonitrile (0.5 mL) was added and the solution was evaporated to dryness again. To the dried $[^{18}F]$fluoride a solution of BMT-180478 in DMSO is added. The reaction vessel is heated for 10 minutes at 120°C. The reaction mixture is diluted with H_2O and purified over a Phenomenex Luna C18 5 µm 250×10 HPLC column. The collected $[^{18}F]$BMT-187144 is trapped on a solid phase extraction (SPE) cartridge. 1.5 mL of ethanol is used to elute $[^{18}F]$BMT-187144 from the SPE cartridge into a reaction vessel after which the ethanol is evaporated to a minimal volume under reduced pressure and at 90°C. After cooling to ambient temperature $[^{18}F]$BMT-187144 is reacted with BMT-192920 in 200 µL PBS for 45 min at 45°C to form the final compound. The crude reaction mixture is diluted till 1 mL with PBS and purified over preconditioned PD-10 desalting Size-Exclusion-Column The

collected fraction containing the [^{18}F]BMS-986229 is diluted in PBS followed by filter sterilization using a 0.22 µM filter using vacuum.

The mean of the product pH was 7.2 ± 0.2. The radiochemical purity as assessed by radioHPLC and Thin layer chromatography (TLC) was 100 and 99.3 ± 0.6%. For radioHPLC a 10 µL product sample was injected and analysed on a Zenix-SEC 300 column (Seppax Technologies) using a mixture of 0.05 M sodium phosphate, 0.15 M sodium chloride (pH 6.8) and 0.01 M NaN_3 as the eluent at a flow rate of 1.0 mL/min. TLC was performed on Merck (Darmstadt, Germany) precoated silica gel 60 F254 plates using 10% methanol in dichloromethane as eluent, where the product BMS-986192 remained on the baseline and the intermediate BMT-187144 runs at the solvent front. Sterility of each [^{18}F]BMS-986192 batch was assured by sterility testing of a sample of the product batch (Bactimm, Nijmegen). These procedures resulted in a sterile final product with endotoxin levels <0.2 EU/mL.

Preparation and QC of ^{89}Zr-Nivolumab

^{89}Zr-Nivolumab has been produced in compliance with current Good Manufacturing Practice at the VU University Medical Center. The procedures of radiolabelling of Nivolumab with ^{89}Zr have been validated with respect quality controls and the final quality of the product. 89Zr-Nivolumab has been produced according to previously reported method of Vosjan et al. [1]. In short, 5 mg nivolumab (10 mg/mL) was diluted with 470 µL 0.9% NaCl. The pH was adjusted to 8.9-9.1 with 0.1 M Na_2CO_3. This solution was added to 20 µL of 5 mM (3 equivalents) NCS-Bz-DFO in DMSO (Macrocyclics, Boston, USA). The solution was shaken in a thermomixer for 30 min at 37˚C at 550 rpm. Next, the conjugated DFO-Nivolumab was purified by size exclusion chromatography (PD10, GE Healthcare) and the product collected in 50 mM NaOAc + 200 mM sucrose pH 5.50±0.30. Finally DFO-Nivolumab was radiolabelled. To this end 200 µL 1M oxalic acid containing the required amount of ^{89}Zr was mixed with 90 µL 2M Na_2CO_3 and reacted for 3 minutes. Next 1 mL 0.5 M Hepes and 0.71 mL DFO-Nivolumab (~1.7 mg) were added and reacted for 60 minutes at room temperature while slowly shaken. After the incubation period ^{89}Zr-Nivolumab was purified by size exclusion chromatography using a PD10 column. The product was eluted in 50 mM NaOAc + 200 mM Sucrose pH 5.50±0.30. The product was formulated to arrive at an injection dose of 37 MBq – 2 mg – 20 mL ^{89}Zr-Nivolumab. The mean of the product pH was 5.78 ± 0.05. The mean radiochemical purity as assessed by spin filter was 99.4 ± 0.6%. To this end 4 µL of product was diluted with 96 µL eluent (5% DMSO and 95% 50 mM NaOAc + 200 mM sucrose buffer) and applied on a microcon-30 centrifugal filter unit (Ultracel YM-30, regenerated cellulose, 30 kDa cut-off, Merck Millipore). The solution was spun down for 7 min at 14000 rpm (Eppendorf 5430). The filter was washed twice with 100 µL eluent and spun down at 14000 rpm for 7 min after each wash step. The filtrate contained free ^{89}Zr/^{89}Zr-DFO, while the radiolabeled mAb was left on the filter. The mean radiochemical purity was 99.4 ± 1.9% and the mean protein integrity was 99.2 ± 1.8% as determined by size exclusion HPLC using a superdex 200 10/30 GL size exclusion column (GE healthcare Life sciences) including a guard column using a mixture of 0.05 M sodium phosphate, 0.15 M sodium chloride (pH 6.8) and 0.01 M NaN_3 as the eluent at a flow rate of 0.5 mL/min.. The mean immune reactive fraction as assessed by a cell binding assay was 88.2 ± 5.0%. Sterility of each ^{89}Zr-nivolumab batch was assured by performing a media fill immediately after final filter sterilisation of each batch. These procedures resulted in a sterile final product with endotoxin levels <0.2 EU/mL.

Supplementary References

1. Vosjan, M.J., *et al.* Conjugation and radiolabeling of monoclonal antibodies with zirconium-89 for PET imaging using the bifunctional chelate p-isothiocyanatobenzyl-desferrioxamine. *Nat Protoc* **5**, 739-743 (2010).
