# Supplementary material for: Whole body PD-1 and PD-L1 positron emission tomography in patients with non-small-cell lung cancer
Source: Nat Commun. 2018 Nov 7;9:4664. doi: 10.1038/s41467-018-07131-y (PMC6220188; doi:10.1038/s41467-018-07131-y)
Supplement: Supplementary file 3 — Reporting Summary [file 41467_2018_7131_MOESM3_ESM.pdf]

nature research

Corresponding author(s): *Double-blind peer review submissions: write DBPR and your manuscript number here instead of author names.*

Reporting Summary

Nature Research wishes to improve the reproducibility of the work that we publish. This form provides structure for consistency and transparency in reporting. For further information on Nature Research policies, see Authors & Referees and the Editorial Policy Checklist.

Statistical parameters

When statistical analyses are reported, confirm that the following items are present in the relevant location (e.g. figure legend, table legend, main text, or Methods section).

n/a	Confirmed	
☐	☒	The exact sample size (*n*) for each experimental group/condition, given as a discrete number and unit of measurement
☐	☒	An indication of whether measurements were taken from distinct samples or whether the same sample was measured repeatedly
☐	☒	The statistical test(s) used AND whether they are one- or two-sided<br>*Only common tests should be described solely by name; describe more complex techniques in the Methods section.*
☒	☐	A description of all covariates tested
☒	☐	A description of any assumptions or corrections, such as tests of normality and adjustment for multiple comparisons
☐	☒	A full description of the statistics including central tendency (e.g. means) or other basic estimates (e.g. regression coefficient) AND variation (e.g. standard deviation) or associated estimates of uncertainty (e.g. confidence intervals)
☒	☐	For null hypothesis testing, the test statistic (e.g. *F*, *t*, *r*) with confidence intervals, effect sizes, degrees of freedom and *P* value noted<br>*Give P values as exact values whenever suitable.*
☒	☐	For Bayesian analysis, information on the choice of priors and Markov chain Monte Carlo settings
☒	☐	For hierarchical and complex designs, identification of the appropriate level for tests and full reporting of outcomes
☐	☒	Estimates of effect sizes (e.g. Cohen's *d*, Pearson's *r*), indicating how they were calculated
☒	☐	Clearly defined error bars<br>*State explicitly what error bars represent (e.g. SD, SE, CI)*

Our web collection on statistics for biologists may be useful.

Software and code

Policy information about availability of computer code

Data collection	Data collection was done in Castor EDC. Castor EDC is a cloud-based Electronic Data Capture platform that enables every researcher worldwide to easily capture high quality, reusable data.
Data analysis	Statistical analysis were performed using SPSS Statistics for windows version 22.0.

For manuscripts utilizing custom algorithms or software that are central to the research but not yet described in published literature, software must be made available to editors/reviewers upon request. We strongly encourage code deposition in a community repository (e.g. GitHub). See the Nature Research guidelines for submitting code & software for further information.

Data

Policy information about availability of data

All manuscripts must include a data availability statement. This statement should provide the following information, where applicable:

- Accession codes, unique identifiers, or web links for publicly available datasets
- A list of figures that have associated raw data
- A description of any restrictions on data availability

The data that support the findings of this study are available from the authors on reasonable request

Field-specific reporting

Please select the best fit for your research. If you are not sure, read the appropriate sections before making your selection.

☒ Life sciences ☐ Behavioural & social sciences ☐ Ecological, evolutionary & environmental sciences

For a reference copy of the document with all sections, see nature.com/authors/policies/ReportingSummary-flat.pdf

Life sciences study design

All studies must disclose on these points even when the disclosure is negative.

Sample size	13
Data exclusions	0
Replication	Not applicable
Randomization	Not applicable
Blinding	Not applicable

Reporting for specific materials, systems and methods

Materials & experimental systems

n/a	Involved in the study	
☒	☐	Unique biological materials
☐	☒	Antibodies
☒	☐	Eukaryotic cell lines
☒	☐	Palaeontology
☒	☐	Animals and other organisms
☐	☒	Human research participants

Methods

n/a	Involved in the study	
☒	☐	ChIP-seq
☒	☐	Flow cytometry
☒	☐	MRI-based neuroimaging

Antibodies

Antibodies used	DAKO 28.8 antibody and Cell Marque Corporation Clone NAT105 (PD-1) antibody
Validation	DAKO 28.8 antibody: https://www.agilent.com/cs/library/brochures/29125_pd-l1-ihc-28-8-pharmdx-brochure_row.pdf and for Cell Marque Corporation Clone NAT105 (PD-1) antibody: Programmed death-1 (PD-1), also known as CD279, is a type-I transmembrane protein expressed on T-cells, B-cells, and monocytes during activation.1-2 PD-1 and its ligands (PD-L1 and PD-L2) function as an immune checkpoint pathway by regulating T-cell activation and autoimmunity.2<br>1.Thibult ML, et al. Int Immunol. 2013; 25:129-37.<br>2.Francisco L, et al. Immunol. 2010; 236:219-42.

Human research participants

Policy information about studies involving human research participants

Population characteristics	Non-small cell lung cancer patients with ECOG performance status of 0 or 1.
Recruitment	Recruitment was done on the outpatient clinic. All patients with stage IV NSCLC were informed about this study and could participate.
